# Application of new biologic disease criteria for synucleinopathies to the systemic synuclein sampling study cohort

**DOI:** 10.1038/s41531-025-00919-y

**Published:** 2025-04-06

**Authors:** L. M. Chahine, M. C. Brumm, A. R. Nair, S. Mosovsky, N. K. Polinski, T. G. Beach, B. Mollenhauer, C. H. Adler, On behalf of the Systemic Synuclein Sampling Study, Rizwan Akhtar, Amy Amara, Vanessa Arnedo, David P. Breen, Michael C. Brumm, Chelsea Caspell-Garcia, Christopher Coffey, John Crary, Kuldip Dave, Tatiana Foroud, Penelope Hogarth, Danna Jennings, Katherine Kopil, Carly Linder, Connie Marras, David Munoz, Luis Oliveira, Lindsey Riley, David Russell, John Seibyl, Geidy E. Serrano, Naomi Visanji, Charles L. White

**Affiliations:** 1 Department of Neurology, University of Pittsburgh, Pittsburgh, PA USA; 2 Department of Biostatistics, University of Iowa College of Public Health, Iowa City, IA USA; 3 The Michael J. Fox Foundation for Parkinson’s Research, New York, NY USA; 4 Banner Sun Health Research Institute, Sun City, AZ USA; 5 Center of Parkinsonism and Movement Disorders, Department of Neurology, Paracelsus-Elena Klinik Kassel and University Medical Center Göttingen, Göttingen, Germany; 6 Department of Neurology, Mayo Clinic College of Medicine, Scottsdale, AZ USA; 7 Ken and Ruth Davee Department of Neurology and Simpson Querrey Center for Neurogenetics, Northwestern University Feinberg School of Medicine, Chicago, IL USA; 8 Department of Neurology, University of Colorado Anschutz Medical Campus, Aurora, CO USA; 9 University of Edinburgh, Edinburgh, UK; 10 Departments of Pathology, Neuroscience, and Artificial Intelligence & Human Health, Icahn School of Medicine at Mount Sinai, New York, NY USA; 11 The ALS Association, Arlington, VA USA; 12 Indiana University, Indianapolis, IN USA; 13 Oregon Health and Science University, Portland, OR USA; 14 Denali Therapeutics Inc., South San Francisco, CA USA; 15 Perelman School of Medicine at the University of Pennsylvania, Philadelphia, PA USA; 16 The Edmond J. Safra Program in Parkinson’s Disease, University Health Network, University of Toronto, Toronto, Ontario Canada; 17 St. Michael’s Hospital, Toronto, ON Canada; 18 Institute for Neurodegenerative Disorders, New Haven, CT USA; 19 Krembil Brain Institute, Toronto, ON Canada; 20 Edmond J Safra Program in Parkinson Disease, Parkinson Foundation Centre of Excellence, Toronto Western Hospital, Toronto, Canada; 21 Department of Laboratory Medicine and Pathobiology, University of Toronto, Toronto, ON Canada; 22 Tanz Centre for Research in Neurodegenerative Diseases, University of Toronto, Toronto, ON Canada; 23 Department of Pathology, University of Texas Southwestern Medical Center, Dallas, TX USA

**Keywords:** Neuroscience, Neurology, Neurodegenerative diseases, Parkinson's disease

## Abstract

We applied biologic criteria and NSD-ISS to Synuclein Sampling Study, a PD sample with range of durations since clinical diagnosis. 93% of evaluable participants met biologic criteria. The majority were NSD-ISS stage3 or less, including >40% of the advanced PD group. Interpretation of staging in medicated patients is challenging. Biologic criteria and clinico-biomarker staging are important steps forward, but quantitative biomarkers of disease progression, not influenced by medications, are critically needed.

We read with interest the article by Dam et al.^1^. There have been two newly proposed biologic criteria for Parkinson’s disease (PD), partially defined by in vivo evidence of pathologic alpha-synuclein (asyn) and neurodegeneration (termed Neuronal α-Synuclein Disease [NSD] by Simuni et al.^2^ and SynNeurGe by Höglinger et al.^3^). In addition to proposing this new biologic definition, Simuni et al. put forth a staging system, the Neuronal α-Synuclein Disease Integrated Staging System (NSD-ISS). In the manuscript by Dam et al., criteria are proposed to define stages 0–6 of the NSD-ISS and they report results of applying these criteria to cohorts of individuals with a clinical diagnosis of early PD.

We have applied the NSD, NSD-ISS, and SynNeurGe to an independent cohort, the Systemic Synuclein Sampling Study (S4)^4–6^. The S4 aimed to examine the distribution of abnormal alpha-synuclein across different tissues and body fluids in PD; the study sample and available data^7^ offer a prime opportunity for the present post-hoc analysis. Unlike the cohorts examined by Dam et al., the S4 sample is comprised of individuals with a range of disease durations and severities, defined as follows: early PD (≤ 2 years since diagnosis, not treated with dopaminergic medication (exceptions made for rasagiline)), moderate PD (>2 but <5 years since diagnosis, treated with dopaminergic medication but without motor fluctuations), and advanced PD (≥5 years since diagnosis, with motor fluctuations). Other enrollment criteria were reduced dopamine transporter binding on SPECT scan (based on visual interpretation) and absence of dementia. Aggregated asyn was assessed in CSF and submandibular gland with asyn seed amplification assay (SAA), and in skin, submandibular gland, and colon with immunohistochemistry, as described^5,6^.

The study enrolled 61 individuals with a clinical diagnosis of PD (20 early, 20 moderate, 21 advanced). CSF was not available from seven (four early, two moderate, one advanced), and four were CSF asyn SAA negative (two moderate, two advanced). None of the participants had known pathogenic variants in LRRK2, GBA, or other PD-associated genes^8^. Thus, 50 individuals with a clinical diagnosis of PD (82% of those enrolled and 93% of those from whom CSF was available) met criteria for NSD (S + D + G−) and SynNeurGe (Syn+Neur+Ge−).

Despite differences in enrollment criteria, the majority of the sample, 33/50 (66%), were NSD-ISS stage 3, similar to the Dam et al. study (Table 1). Half of the advanced PD group were stage 4, but two (13%) of the early PD group were stage 4 as well, consistent with findings by Dam et al. that a recent diagnosis is not a sufficient indicator of early/mild disease severity.

**Table 1:** Characteristics and NSD-ISS stages of S4 study participants who met NSD and SynNeurGe criteria, according to enrollment cohort

	All PD (*N* = 50)	Early PD (*N* = 16)	Moderate PD (*N* = 16)	Advanced PD (*N* = 18)	p-value
Age, mean (SD) years	63.1 (8.4)	62.0 (9.7)	59.4 (5.3)	67.4 (7.8)	0.0054
Time since diagnosis, mean (SD) years	4.8 (4.6)	0.8 (0.7)	3.6 (1.2)	9.4 (4.4)	<0.0001^a^
LEDD, mean (SD) mg	460.0 (443.0)	37.5 (61.9)	405.3 (182.9)	884.2 (414.2)	<0.0001
NSD-ISS Stage					0.0056^b^
Stage 2B, N (%)	4 (8%)	2 (13%)	1 (6%)	1 (6%)	
Stage 3, N (%)	33 (66%)	12 (75%)	14 (88%)	7 (39%)	
Stage 4, N (%)	12 (24%)	2 (13%)	1 (6%)	9 (50%)	
Stage 5, N (%)	1 (2%)	0	0	1 (6%)	

*LEDD* Levodopa equivalent dosage.

^a^PD groups were recruited based on time since diagnosis.

^b^In pairwise comparison, stages varied significantly between the moderate and advanced PD (*p*-value = 0.0036) and between the early and advanced PD (*p*-value = 0.0221). Stages 4 and 5 were combined into single category for Fisher’s exact test due to low counts.

It is notable that >40% of the advanced PD group were in stage 3 or less. Given that all advanced PD participants were treated with dopaminergic medication, interpretation of staging in these medicated patients is challenging. It is certainly possible that some individuals with PD of many-years duration still have mild disease. However, two of the core assessments proposed by Dam et al. for the definition of the stages in the NSD-ISS are susceptible to medication effects: presence/absence of parkinsonism, and the Movement Disorders Society Unified Parkinsons Disease Rating Scale (MDS-UPDRS) part II (which ascertains function “on average” rather than specifically determining function in medication OFF or ON states). An examination of a selected case illustrates this point. One participant enrolled in the advanced group was a female aged 70–74 years. Despite a disease duration of 8.3 years since diagnosis, the results of the patient and clinician-reported outcomes indicate relatively mild disease, with minimal functional impairment: Schwab and England score were 90, MDS-UPDRS part III score was 18 in the medication OFF state and 12 in the medication ON state, and Montreal Cognitive Assessment (MoCA) was 30. However, this participant was being treated with 660 mg of levodopa equivalents, and the extent to which the functional level is a result of medication versus underlying disease severity simply cannot be ascertained.

Four participants enrolled in the S4 PD cohort had a negative CSF asyn SAA, despite reduced DAT binding on SPECT scan. While S4 measured pathologic asyn in multiple tissues and biofluids^5,6^, none of the participants who had negative CSF had abnormal asyn assessed with other assays endorsed in the SynNeurGe criteria^3^. Thus, these individuals have parkinsonism and nigral degeneration without evidence of aggregated asyn. Whether these individuals have false negative results, have a form of pathologic asyn undetected by the methods used, or have an underlying biology that is completely independent of asyn, is unknown at this time.

As for the NSD-ISS, results of application to a medicated cohort such as S4 emphasize the need for quantitative biomarkers of disease progression that are not influenced by medications. This will be critical before a staging system can be put forth that will be useful and widely applicable in research and the clinic without the confounds of symptomatic therapies.

## Data Availability

Data for this study can be accessed at: Systemic Synuclein Sampling Study. *Zenodo MJFF Data Repository*; https://zenodo.org/records/7431680 (2022).
